# F46. LUMATEPERONE (ITI-007): FAVORABLE SAFETY PROFILE IN AN OPEN LABEL SAFETY SWITCHING STUDY FROM STANDARD-OF-CARE ANTIPSYCHOTIC THERAPY IN PATIENTS WITH SCHIZOPHRENIA

**DOI:** 10.1093/schbul/sby017.577

**Published:** 2018-04-01

**Authors:** Robert Davis, Alex Dmitrienko, Steven Glass, Susan Kozauer, Jelena Saillard, Michal Weingart, Andrew Satlin, Sharon Mates, Christoph Correll, Kimberly Vanover

**Affiliations:** 1 Intra-Celluar Therapies, Inc; 2 Mediana Research; 3 The Zucker Hillside Hospital, Hofstra North Shore LIJ School of Medicine

## Abstract

**Background:**

Lumateperone (ITI-007) is a first-in-class investigational agent in development for the treatment of schizophrenia. Acting synergistically through serotonergic, dopaminergic and glutamatergic systems, lumateperone represents a new approach to the treatment of schizophrenia and other neuropsychiatric disorders. Lumateperone is a potent antagonist at 5-HT2A receptors and exhibits serotonin reuptake inhibition. Lumateperone also binds to dopamine D1 and D2 receptors acting as a mesolimbic/mesocortical dopamine phosphoprotein modulator (DPPM) with pre-synaptic partial agonism and post-synaptic antagonism at D2 receptors and as an indirect glutamatergic (GluN2B) phosphoprotein modulator with D1-dependent enhancement of both NMDA and AMPA currents via the mTOR protein pathway. Lumateperone demonstrated antipsychotic efficacy in two well-controlled clinical trials and was found to be well tolerated with a safety profile similar to placebo in all trials conducted to date.

**Methods:**

In an open-label safety study, 302 patients with schizophrenia were switched from standard-of-care (SOC) antipsychotic therapy to 6 weeks treatment with lumateperone (ITI-007 60 mg, equivalent to 42 mg active base) QPM with no dose titration, then switched back to SOC for 2 weeks. The primary objective was to determine the safety of lumateperone, assessed by adverse events, body weight, 12-lead electrocardiograms, vital signs, clinical laboratory tests, motor assessments, and the Columbia-Suicide Severity Rating Scale. The secondary objectives were to determine the effectiveness of lumateperone to improve psychopathology as measured by the PANSS, social functioning as measured by the PANSS Pro-Social Factor and the Personal and Social Performance Scale (PSP), and depression as measured by the Calgary Depression Scale for Schizophrenia.

**Results:**

Lumateperone was generally well-tolerated with a favorable safety profile. There was no drug related serious adverse event. In comparison to treatment with SOC antipsychotics at baseline, mean body weight decreased with lumateperone treatment. Lumateperone also demonstrated a favorable cardiometabolic and endocrine safety profile. Mean levels of cholesterol, triglycerides and prolactin improved with lumateperone treatment and worsened again when patients returned to SOC. The cardiovascular safety of lumateperone was favorable including no QTc interval prolongation. While efficacy data in an open-label study should be interpreted cautiously due to the absence of a parallel control group, improvements were observed in change from baseline of the PANSS total scores. Improvements were also seen in the Positive symptom subscale score, General Psychopathology subscale score, Marder Negative Factor score, and Prosocial Factor score as well as in the PSP scale. Greater improvements were observed in subgroups of patients with elevated symptomatology such as those with comorbid symptoms of depression and those with prominent negative symptoms.

**Discussion:**

Lumateperone represents a novel approach to the treatment of schizophrenia with a favorable safety profile. The lack of metabolic, motor and cardiovascular safety issues presents a safety profile differentiated from standard-of-care antipsychotic therapy. Patients with stable symptoms on other antipsychotics may further improve when switched to lumateperone, with no dose titration needed. These data may warrant further investigation in placebo-controlled trials in patients with prominent negative symptoms and, separately, in patients with comorbid depression to demonstrate efficacy in these populations.

